# Antisaccades in Parkinson’s Disease: A Meta-Analysis

**DOI:** 10.1007/s11065-021-09489-1

**Published:** 2021-03-19

**Authors:** Josefine Waldthaler, Lena Stock, Justus Student, Johanna Sommerkorn, Stefan Dowiasch, Lars Timmermann

**Affiliations:** 1 Department of Neurology, University Hospital Marburg, 35033 Marburg, Germany; 2 CMBB – Center for Mind, Brain and Behavior, Universities Gießen and Marburg, Marburg, Germany; 3 Department of Neurophysics, University of Marburg, Marburg, Germany; 4 Thomas RECORDING GmbH, Giessen, Germany

**Keywords:** Parkinson’s disease, Antisaccade, Saccade, Eye movement, Eye-tracking, Executive functions

## Abstract

The usefulness of eye-tracking tasks as potential biomarkers for motor or cognitive disease burden in Parkinson’s disease (PD) has been subject of debate for many years. Several studies suggest that the performance in the antisaccade task may be altered in patients with PD and associated with motor disease severity or executive dysfunction. In this meta-analysis, random effects models were used to synthesize the existing evidence on antisaccade error rates and latency in PD. Furthermore, meta-regressions were performed to assess the role of motor and cognitive disease severity, dopaminergic medication and methodological factors. Additionally, the impact of acute levodopa administration and activation of deep brain stimulation was evaluated in two separate sub-analyses.

This meta-analysis confirms that antisaccade latency and error rate are significantly increased in PD. Disease duration, Unified Parkinson’s disease rating scale score and Hoehn and Yahr stage mediate the effect of PD on antisaccade latency with higher motor burden being associated with increased antisaccade latency.

Acute administration of levodopa had no significant effects on antisaccade performance in a small number of eligible studies. Deep brain stimulation in the subthalamic nucleus, on the other hand, may alter the speed accuracy trade-off supporting an increase of impulsivity following deep brain stimulation in PD.

According to the results of the meta-analysis, antisaccade latency may provide a potential marker for disease severity and progression in PD which needs further confirmation in longitudinal studies.

**Supplementary Information:**

The online version contains supplementary material available at 10.1007/s11065-021-09489-1.

## Introduction

While James Parkinson stated in his original description of Parkinson’s disease (PD) that cognition was preserved in PD, cognitive impairment, in particular a dysexecutive syndrome, is nowadays regarded as a central symptom that PD patients may present even in the earliest stages of the disease. (Kudlicka et al., 2011) As part of the executive functions, inhibition control describes the ability to suppress habitual or prepotent reflexive responses to stimuli. (Obeso et al., 2011). Impaired response inhibition may result in an increased impulsivity, i.e. a tendency to act without delay, reflection or voluntary directing (Bari & Robbins, 2013). One of the successfully validated experimental paradigms used to explore inhibition of prepotent reflexive responses is the *antisaccade task*. (Everling & Fischer, 1998) In this task, participants are instructed to look at the opposite direction of the presented target stimulus. To successfully perform an antisaccade, a faster reflexive, visually-guided saccade in the direction of the stimulus has to be suppressed and consecutively a voluntary saccade to the opposite direction has to be planned and executed. The error rate in the antisaccade task has been associated with executive functions in healthy individuals as well as several in neurodegenerative disorders. (Mirsky et al., 2011; Rodríguez-Labrada et al., 2014; Walton et al. 2015) Please see also (Leigh et al., 2015) for a comprehensive review on the neural basis of saccade control.

Eye-tracking studies are especially suitable to assess cognitive functions in movement disorders like PD, as they do not rely on a motor response (e.g. pressing a button or pulling a lever) and, thus may be less influenced by bradykinesia in PD. However, the results of studies assessing antisaccade performance in PD patients are inconsistent, sometimes even contradictory. While no meta-analysis on the effect of PD on antisaccades has been published so far, alterations of visually-guided saccades in PD have been reviewed in a meta-analysis in 2010 (Chambers & Prescott, 2010): Chambers and colleagues concluded that, overall, the latencies of visually-guided saccades are prolonged in PD. However, the latency may differ as a function of target amplitude, with small amplitudes resulting in “hyper-reflexive” saccades with decreased latencies and larger amplitudes resulting in prolonged latencies compared to healthy subjects. Hence, the high variability in task-related variables might also contribute to the inconsistent findings in antisaccade studies in PD.

While some studies demonstrated increased antisaccade error rates even in unmedicated patients in very early disease stages (Antoniades et al., 2015a; Hanuška et al. 2019), others found no significant alterations of antisaccade latency or antisaccade error rate early and later in the disease course. (Ranchet et al., 2017; Nagai et al., 2019) As proposed by two recent studies (Lu et al., 2019; Waldthaler et al., 2019a), antisaccade latency might correlate with disease duration in PD without significant impact of dopaminergic medication.

Surprisingly few studies, most with relatively small sample sizes, assessed treatment effects on antisaccades in PD so far. Dopaminergic replacement therapies as well as deep brain stimulation in the subthalamic nucleus (STN-DBS) and the internal segment of globus pallidus (GPi-DBS) might influence antisaccade performance differently and via distinct mechanisms. It has been proposed that levodopa intake may increase the latency of reflexive saccades, while it might decrease the latency of voluntarily executed saccades, like antisaccades. (Terao et al., 2013) Recently, Lu et al. supported this finding in their meta-analysis on the effect of levodopa on saccades. However, they did not replicate the decreasing effect of levodopa on antisaccade latency in their own study. (Lu et al., 2019) While antisaccade error rates might decrease after levodopa intake (Hood et al., 2007), STN-DBS studies provided inconsistent results regarding changes in antisaccade error rates. (Antoniades et al., 2015b; Goelz et al., 2017)

To shed light upon the high variability of antisaccade performance in PD, we conducted a comprehensive meta-analysis. The two most frequently reported variables were selected as principal outcome measures for antisaccade performance: the error rate and the latency. The aims of this meta-analysis were (1) to identify the extent of antisaccade deficits in PD patients compared to healthy individuals, (2) to determine the effect of levodopa medication and STN-DBS on antisaccade performance in PD and (3) to investigate clinical factors and task related variables that moderate antisaccade performance in PD.

## Methods

### Search Strategy

**Fig. 1:**
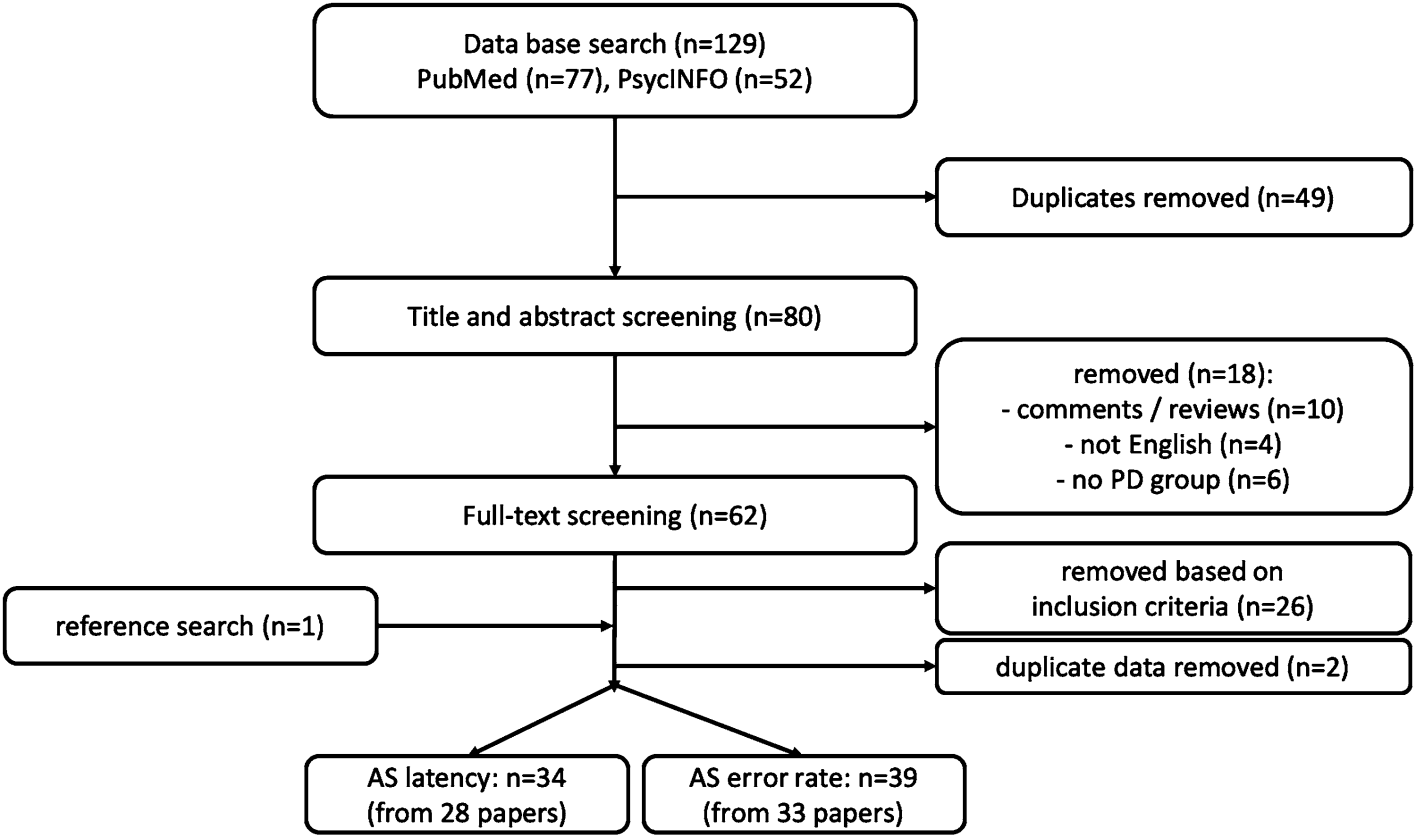
Flowchart of the literature search procedure

The meta-analysis was conducted in accordance with the recommendations of the *Meta-Analysis of Observational Studies in Epidemiology Group, the Preferred Reporting Items for Systematic Reviews and Meta-Analyses* (PRISMA) 2009 guidelines (Stewart et al., 2015) and the review protocol was submitted to PROSPERO (registration number: CRD42020204579). The systematic literature review was performed in April 2020. The search terms “("Parkinson" OR "Parkinson's”) AND (“antisaccade” OR "antisaccade”)” yielded a total of 129 results on PsycINFO (*n*=52) and PubMed (*n*=77). After removal of duplicates, titles and abstracts of the remaining 80 records were screened by two authors separately (LS, JS or JSt and JW) to exclude editorials, comments, books and review papers as well as studies that did not report results from patients with PD. All full texts that were not excluded based on those criteria were screened for fulfillment of the following pre-defined inclusion criteria: Full length original research articles in English published until March 31^st^, 2020 which reported results of either a human PD group and a healthy control group or a PD group in two treatment states (on and off medication or on and off DBS) that performed an antisaccade task using any eye-tracking device. No restrictions regarding disease stage, cognitive impairment, age, or gender were defined. When identical data sets were reported in more than one paper (for example Goelz et al., 2017, 2019), the record with the earliest publication date was selected. When subjects performed more than one task design within one publication, both were included into the meta-analysis as separate studies. Additionally, the references of all publications were screened to identify further studies that were missed by the literature search, which identified one new study. In total, 42 records fulfilled the inclusion criteria. Please see Fig. 1 for the flowchart for a complete overview of the search strategy.

**Fig. 2:**
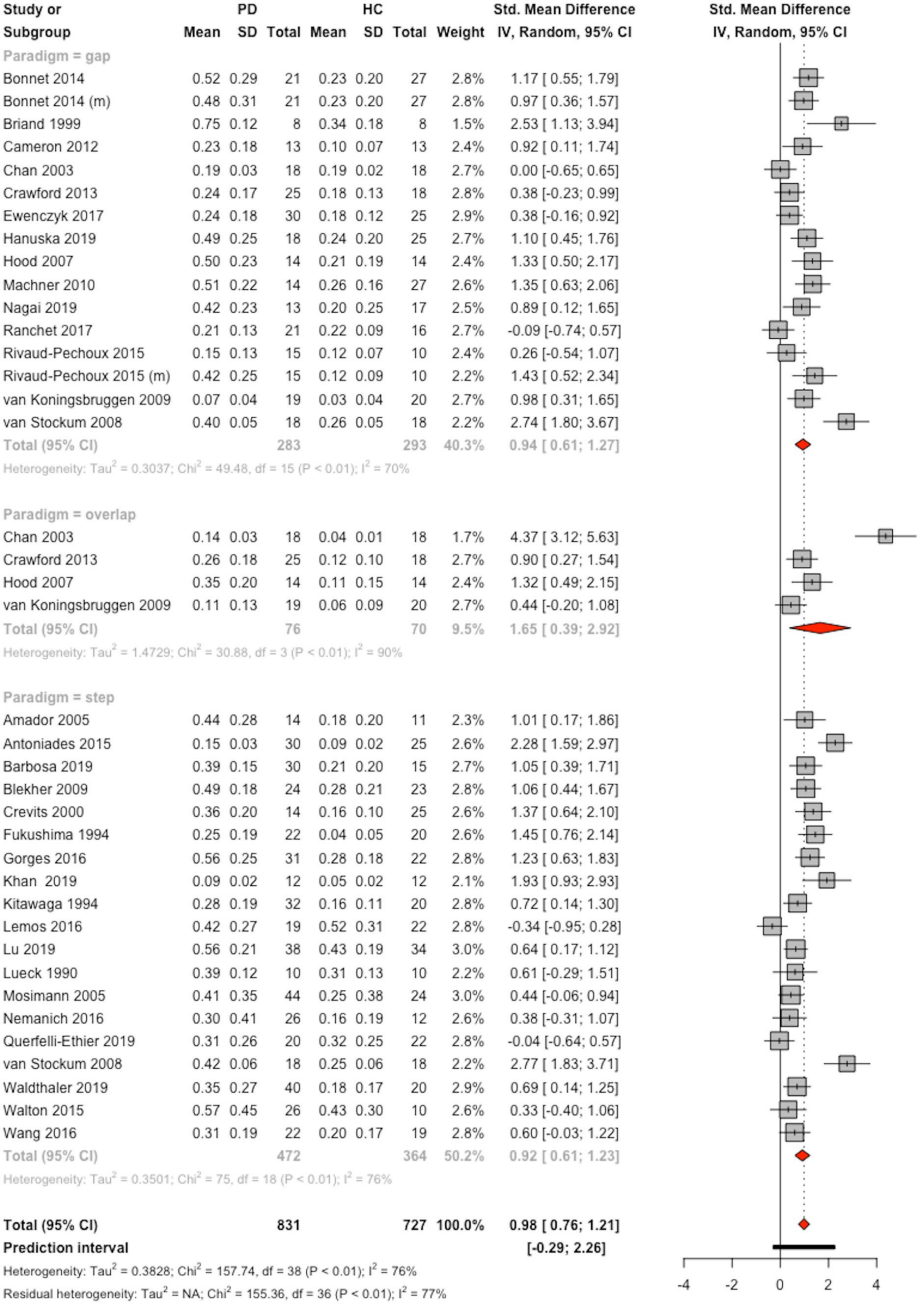
Forest plot for antisaccade error rate, sorted by paradigm with studies in alphabetical order. The size of the grey box represents the weight of the study in the meta-analysis. *CI* confidence interval, *HC* healthy control group, *PD* group with Parkinson’s disease, *(m)* mixed task design with prosaccades and antisaccades, *SD* standard deviation, *Std.* standardized

Records were simultaneously screened for studies that reported results from the same PD group in two medication states or two STN-DBS states (on and off). Regarding levodopa, four papers with five studies were identified, including a total of 119 subjects for antisaccade latency and antisaccade error rate. Eight eligible STN-DBS studies were identified with a total of 66 subjects for antisaccade error rate and 89 subjects for antisaccade latency. We refrained from a meta-analysis of GPi-DBS effects as only two studies were identified for this DBS target.

### Data Extraction

Data extraction and quality assessment were conducted by two independent investigators (LS, JSt or JS and JW). Besides mean and standard deviation (SD) of antisaccade error rate and antisaccade latency, the following information were extracted from each study: name of the first author, publication year, mean age, mean levodopa equivalent daily dosage, mean disease duration, mean Hoehn & Yahr stage (Hoehn & Yahr, 1967), mean Montreal Cognitive Assessment score (MoCA), mean Mini Mental State Examination score (MMSE), exclusion of PD-dementia, proportion of patients tested in on medication state.

The exclusion of dementia criterion was assigned when a) the study explicitly stated the exclusion of PD-dementia or b) the study used an MMSE or MoCA cut-off score to exclude cognitively impaired subjects, albeit we acknowledge that using a cut-off score of a global cognitive screening tool is not sufficient to accurately diagnose or exclude dementia. (Emre et al., 2014)

To acknowledge the potential impact of the large methodologic variety in task designs, several task-related factors were extracted: paradigm design (step, gap or overlap task; antisaccade-only task or randomly mixed task of antisaccades and visually-guided saccades within the same blocks), duration of fixations between trials (fixed or random duration), and target amplitude.

To account for the fact that several studies (36%) included subsets of patients in on and off medication into the same group, medication state is reported as proportion of subjects in on medication state with a range from 0 (all patients were off levodopa for 12 hours or more) to one (all patients were tested after administration of dopaminergic medication). When a study provided results in on and off medication state, antisaccade latency, antisaccade error rates and the corresponding UPDRS scores or Hoehn & Yahr stages in off state were included into the primary meta-analysis. When results were presented as figures, descriptive statistics were extracted using WebPlotDigitizer, version 4.2. (Rohatgi, 2015) For studies that did not provide mean or SD, but other descriptive statistics (i.e., median, minima and maxima or interquartile range), means and SD were estimated using the equations described by Hozo et al. (Hozo et al., 2005) When ranges were reported for the clinical measures, the mean of the range was used as an estimate.

### Statistical Analysis

The meta-analyses and additional statistical analyses were performed using the *metafor* package (Viechtbauer, 2010) in R (R Core Team, 2014). Effect sizes, 95% confidence intervals, variance, standard errors and statistical significance were calculated using a random-effects model. Effect sizes are reported as biased corrected standardized mean difference (Hedges' g) and were pooled across studies for obtaining an overall effect size using the inverse-variance method. To assess heterogeneity among studies, Cochran's Q and Higgins and Green's I^2^ statistics were computed. (Huedo-Medina et al., 2006) Additionally, analysis of publication bias was performed since published studies may provide larger mean effect sizes than unpublished data. (Rothstein et al., 2006) Publication bias was visualized by funnel plots and symmetry, which was detected by Egger’s test. (Egger et al., 1997) If a statistically significant publication bias was detected, the trim-and-fill method was used to adjust for the bias. (Duval & Tweedie, 2000)

Meta-regressions were performed to assess the potentially important clinical and task-related moderators that might explain heterogeneity across the studies for which the respective information was available (see Table 2). MoCA was excluded from the moderator analysis on antisaccade latency because the score was available for less than 10 studies. (Borenstein, 2009) Additionally, the differences between the PD group and healthy control group in age and MMSE score were used as indicators for the quality of the studies’ matching procedure.

A *p*-value of <0.05 was set as the cut-off for significance in all analyses. P-values were adjusted using permutation tests with 1000 permutations implemented in the metafor package to address multiple testing in the meta-regressions. (Higgins & Thompson, 2004) Here, the p-value for the model’s coefficients equals the proportion of times that the absolute value of the test statistic for the coefficient under the permuted data is as extreme or more extreme than under the actually observed data. Permutation-based confidence intervals of the model coefficients were calculated by computationally shifting the observed effect sizes to find the most extreme values for which the permutation test leads to non-rejection.

Two separate meta-analyses were conducted using an identical approach to calculate overall effect sizes of levodopa medication for studies in which the same patient population was tested in on and off medication state, respectively on and off DBS state.

## Results

### Demographic and Clinical Characteristics of the Study Samples

The main characteristics of the selected studies are shown in Table 1. The total sample size was 703 patients with PD and 600 healthy control for antisaccade latency (*k*=34), respectively 831 patients and 727 healthy control for antisaccade error rate (*k*=39). Mean ages comprised between 52 and 77 years, with mean disease duration between 0.7 and 14.7 years and mean UPDRS III scores ranging from five to 85.

**Table 1:** Demographic and clinical characteristics of included studies in alphabetical order

Study	Number of PD patients	Number of healthy controls	paradigm	Target amplitude in °	Blocks	Intervall fixation-target onset	Mean age PD	Mean age HC	Proportion on med.	Mean LEDD	Mean disease duration	Mean UPDRS III	Mean Hoehn & Yahr stage	Mean MoCA	Mean MMSE
(Amador et al., 2006)	14	11	step	7	AS	random	60	55	0	NA	12	85	3.6	NA	NA
(Antoniades et al., 2015a)	30	25	step	NA	AS	random	68	67	0	0	0.7	23	NA	NA	28
(Barbosa et al., 2019)	30	15	step	15	AS	fixed	54	53	1	821	9.9	NA	1.9	29	NA
(Blekher et al., 2009)	24	23	step	15	AS	random	58	59	0.75	NA	NA	28	2.3	NA	29
(Bonnet et al., 2014a)	21	27	gap	13	AS, PSAS	random	55	36	0.95	628	9.6	26	2	NA	28
(Briand et al., 1999)	8	8	gap	7	AS	fixed	74	73	0.13	NA	8.5	45	2.4	NA	27
(Cameron et al., 2013)	13	13	gap	7	PSAS	fixed	65	65	0	369	3.2	24	2.3	NA	29
(Chan et al., 2005)	18	18	gap, overlap	20	AS	fixed	67	66	1	NA	NA	NA	2	NA	NA
(Chen et al., 1999)	11	8	step	15	AS	random	60	57	NA	NA	NA	NA	NA	NA	NA
(Crawford et al., 2013)	25	18	gap, overlap	4	AS	fixed	63	75	0.92	NA	NA	NA	2.1	NA	NA
(Crevits et al., 2000)	14	25	step	14	AS	fixed	70	72	1	NA	NA	20	3.5	NA	28
(Ewenczyk et al., 2017)	30	25	gap	25	AS	random	60	60	1	712	8.8	16	2.3	NA	28
(Fukushima et al., 1994)	22	20	step	15	AS	fixed	57	57	0.68	NA	3.3	NA	NA	NA	NA
(Gorges et al., 2016)	31	22	step	13	AS	random	71	68	1	380	6	12	NA	NA	28
(Hanuška et al., 2019)	18	25	gap	12	AS	random	63	66	0	0	1.6	33	NA	24	NA
(Harsay et al., 2010)	20	18	step	12	AS	random	62	69	0.95	NA	7	17	NA	NA	NA
(Hood et al., 2007)	14	14	gap, overlap	7	AS	random	60	58	0	NA	14.7	56	3.6	NA	28
(Khan et al., 2019)	12	12	step	16	AS	fixed	68	63	1	NA	NA	NA	NA	NA	NA
(Kitagawa et al., 1994)	32	20	step	8	AS	random	58	57	0.72	NA	NA	NA	NA	NA	NA
(Lemos et al., 2016)	19	22	step	10	AS	fixed	67	68	0	690	4	19	1.5	NA	29
(Lu et al., 2019)	38	34	step	10	AS	random	65	65	0	507	2.9	25	1.7	27	29
(Lueck et al., 1990)	10	10	step	11	AS	fixed	66	70	0.9	850	6.4	NA	2.3	NA	NA
(MacHner et al., 2010)	14	27	gap	10	AS	fixed	52	51	NA	NA	NA	22	NA	NA	NA
(Mosimann et al., 2005)	44	24	step	16	AS	random	77	75	0.73	NA	5.8	33	NA	NA	24
(Nagai et al., 2019)	13	17	gap	8	AS	fixed	70	73	NA	364	8.2	NA	2.7	NA	27
(Nemanich & Earhart, 2016)	26	12	step	15	PSAS	random	68	72	0	867	6.7	38	NA	26	NA
(Ouerfelli-Ethier et al., 2018)	20	22	step	8	PSAS	fixed	67	66	0.9	NA	NA	NA	2.1	26	29
(Ranchet et al., 2017b)	21	16	gap	28	AS	fixed	70	61	0.95	382	5.3	29	2	24	NA
(Rivaud-Péchoux et al., 2007)	15	10	gap	25	AS, PSAS	random	64	64	1	NA	6.6	15	NA	NA	NA
(van Koningsbruggen et al., 2009)	19	20	gap, overlap	9	AS	random	67	66	1	66	7.1	15	NA	NA	NA
(van Stockum et al., 2008)	18	18	gap, step	11	AS	random	66	66	0.94	NA	8.8	NA	2	NA	NA
(Visser et al., 2019)	21	19	step	6	AS	random	65	64	0.76	NA	6	17	NA	NA	29
(Waldthaler et al., 2019b)	40	20	step	20	AS	fixed	66	66	0	528	4.9	38	2.5	25	NA
(Walton et al., 2015)	26	10	step	10	PSAS	random	68	64	1	632	8.1	5	2.5	28	30
(Wang et al., 2016)	22	19	step	10	PSAS	fixed	67	69	1	685	6.1	30	2.4	27	NA

*PD* Parkinson’s disease, *HC* healthy controls, *proportion on med.* proportion of PD patients tested in on medication state, *UPDRS III* part III of the Unified Parkinson’s Disease Rating Scale, *MoCA* Montreal Cognitive Assessment, *MMSE* Mini Mental State Examination, *AS* task that included antisaccade blocks only, *PSAS* mixed paradigms that included antisaccade and visually-guided saccades within the same block in randomized order

61% of studies considered PD-dementia an exclusion criterion, while 14 % did not include any statement regarding the handling of cognitive impairment. Out of the 24 studies specifically excluding demented subjects, nine studies did not report the exact clinical criteria for diagnosis of dementia and 15 used a cut-off score of a global cognitive screening test (MMSE or MoCA). Only one study explicitly included a proportion of 50% patients with PD-dementia. (Mosimann et al., 2005) Exploratory exclusion of this study from further analysis did not change the results of this meta-analysis.

### Meta-Analysis: Antisaccade Error Rate

The PD group showed a significantly higher error rate (0.34, 95%-CI=[ 0.30; 0.39]) than the healthy control group (0.19, 95%-CI=[0.16; 0.22]) with an overall effect size of 0.99 (*k*=39, 95%-CI=[ 0.76; 1.21], *t*=8.52, *p*<0.0001).

The prediction interval indicates that 95% of future studies may find an effect size ranging between -0.29 and 2.26 based on the results of the studies included in this meta-analysis (Fig. 2).

Heterogeneity between studies was high (I^2^ = 75.9%, 95%-CI=[67.3%; 82.3%]; H = 2.04, 95%-CI=[1.75; 2.37], Q(df=38)=157.7, *p*<0.0001), and Egger’s test was significant (intercept 6.017, t=4.64, p<0.0001). Therefore, nine virtual studies were added using the trim-and-fill-method which resulted in a persisting significant effect of PD on the antisaccade error rate (*k*=48, g=0.68, 95%-CI=[ 0.42; 0.93], *t*=5.14 *p*<0.0001, see Supplementary Material for the funnel plot).

### Meta-Analysis: Antisaccade Latency

**Fig. 3:**
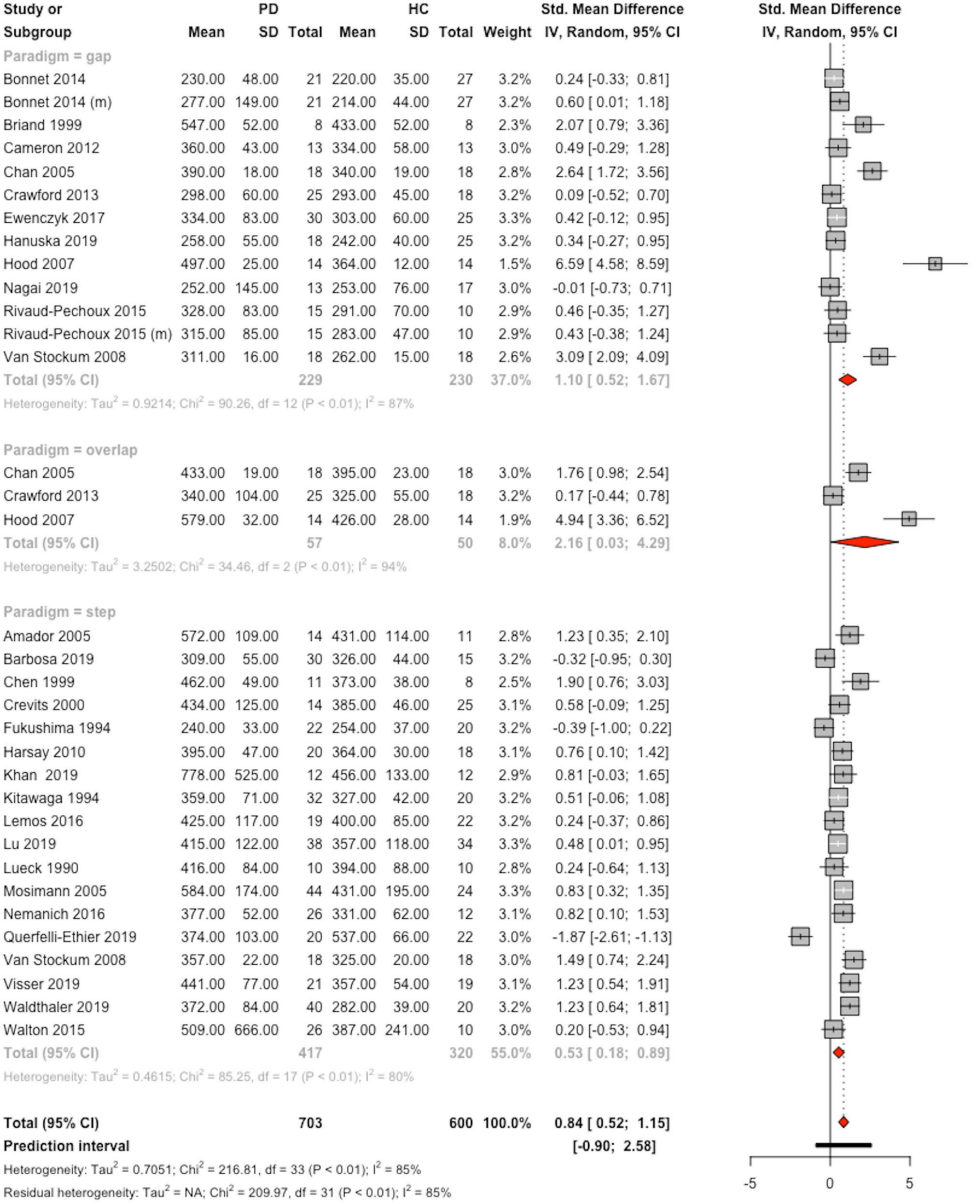
Forest plot for antisaccade latency, sorted by paradigm with studies in alphabetical order. The size of the grey box represents the weight of the study in the meta-analysis. *CI* confidence interval, *HC* healthy control group, *PD* group with Parkinson’s disease, *(m)* mixed task design with prosaccades and antisaccades, *SD* standard deviation, *Std.* standardized

PD had a significantly prolonging effect on antisaccade latency with an effect size of 0.84 (*k*=34, 95%-CI=[0.52; 1.15] *t*=5.24, *p*<0.0001) (Fig. 3). Based on the calculated prediction interval, 95% of future studies may find an effect size ranging between -0.90 and 2.58 (Fig. 3).

Heterogeneity between studies was high (I^2^ = 84.8% 95%-CI=[79.7%; 88.6%]; H = 2.56, 95%-CI=[2.22; 2.96]; Q(df=33)=216.8, *p*<0.0001) and Egger’s test was significant (intercept 6.038 t=4.225, p<0.0001). After adding seven virtual studies using the trim-and-fill-method, the effect remained significant (*k*=41, g=0.41, 95%-CI=[0.059; 0.77], *t*=2.29, *p*=0.02, see Supplementary Material for the funnel plot). The mean latency was 339.8 ms in the PD group and 294.2 ms in the healthy control group in the gap paradigm, respectively 411.7 ms in the PD group and 368.6 ms in the healthy control group in the step paradigm.

### Moderator Analysis

**Fig. 4:**
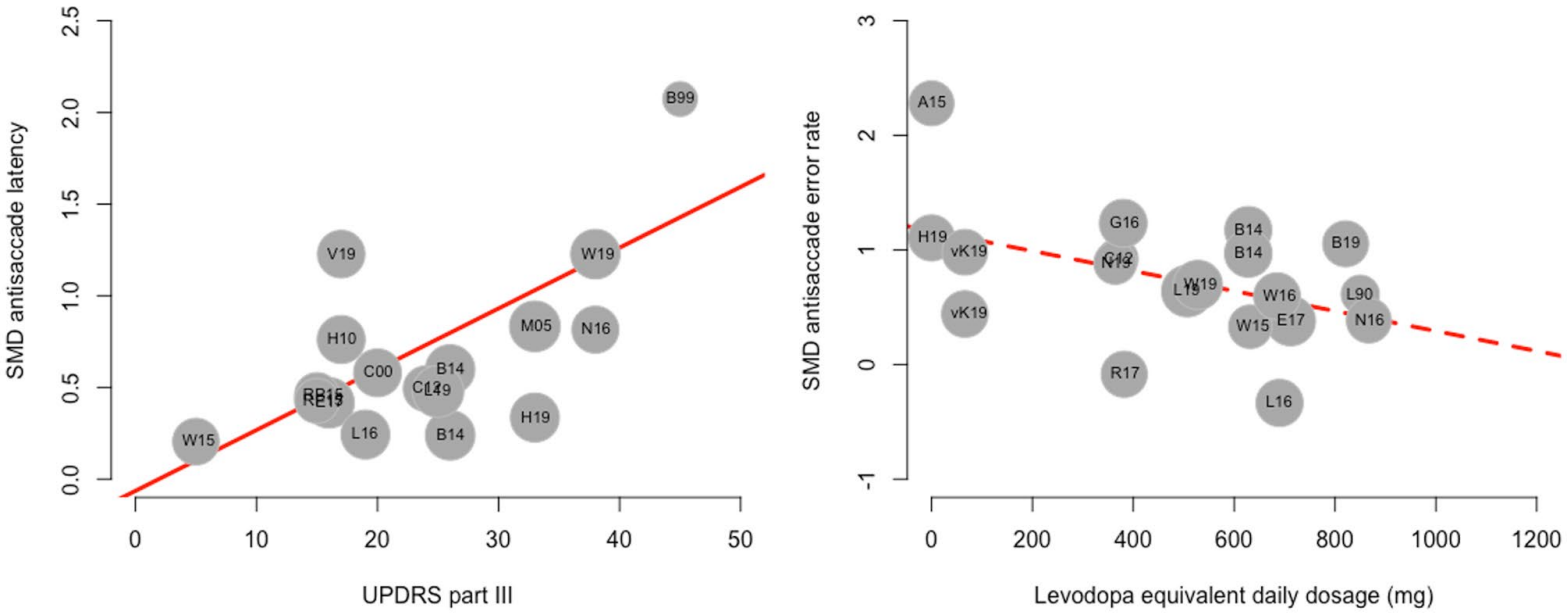
Bubble plots of the mediating / moderating effects of A: mean UPDRS III score on antisaccades latency and B: mean LEDD on antisaccades error rate. *B99* (Briand et al., 1999), *B14 *(Bonnet et al., 2014b), *B19 *(Barbosa et al., 2019), *C12 *(Cameron et al., 2012), *C00 *(Crevits et al., 2000), *E17 *(Ewenczyk et al., 2017), *G16 *(Gorges et al., 2016), *H10 *(Harsay et al., 2010), *H19 *(Hanuška et al., 2019), *L19 *(Lu et al., 2019), *L90 *(Lueck et al., 1990), *L16 *(Lemos et al., 2016), *M05 *(Mosimann et al., 2005), *N16 *(Nemanich & Earhart, 2016), *N19 *(Nagai et al., 2019), *R17* (Ranchet et al., 2017), *RP15 *(Rivaud-Péchoux et al., 2007), *V19 *(Visser et al., 2019), *vK09 *(van Koningsbruggen et al., 2009), *W15 *(Walton et al., 2015), *W16 *(Wang et al., 2016), *W19 *(Waldthaler et al., 2019a)

Meta-regressions revealed that disease duration, Hoehn & Yahr stage and UPDRS III score were mediators of the effect of PD on antisaccade latency (Fig. 4a). PD patients with more severe disease burden executed antisaccades with increasingly higher latency compared to the healthy control group. UPDRS III and disease duration accounted for 26 %, respectively 7% of the heterogeneity across studies.

The levodopa-equivalent daily dosage showed a trend towards a negatively moderating effect on antisaccade error rate with a statistical significance at the cut-off value (*p*=0.05). (Fig. 4b). See Table 2 for the results of all meta-regressions.

**Table 2:** Results of the meta-regressions with permutation test-adjusted p-values and confidence intervals

	Antisaccade error rate						Antisaccade latency					
Moderator	k	R^2^	beta	min 95% CI	max 95% CI	p	k	R^2^	beta	min 95% CI	max 95% CI	p
age difference PD-HC	39	0	0.01	-0.04	0.052	0.69	34	0	0.01	-0.07	0.07	0.79
MMSE difference PD- HC	14	1.8	-0.11	-0.20	0.45	0.45	12	0	0.19	-0.24	NA	0.50
proportion on medication	37	0	0.00	-0.58	0.69	0.99	32	0	-0.78	-1.67	0.20	0.11
age PD group	39	0	-0.01	-0.05	0.04	0.72	34	0	0.00	-0.09	0.08	0.99
levodopa-equivalent daily dose	19	22.0	-0.00	-0.00	0.00	0.05	13	0	0.00	-0.00	0.00	0.78
disease duration	29	0	0.03	-0.06	0.11	0.45	25	6.9	0.22	0.10	0.37	0.001
UPDRS III	26	0	0.01	-0.01	0.02	0.30	20	25.8	0.03	0.01	0.06	0.01
Hoehn & Yahr stage	26	2.9	0.33	-0.54	0.79	0.48	23	2.2	1.15	0.04	2.31	0.02
MoCA	10	0	0.07	-0.14	0.28	0.42	8	NA	NA	NA	NA	NA
MMSE	17	1.6	-0.15	-0.39	0.12	0.25	15	0	-0.18	-0.85	0.64	0.54
exclusion of PD-dementia	39	0	0.28	-0.39	0.64	0.66	34	0	-0.07	-0.86	0.79	0.87
mixed blocks (AS+PS)	39	0.6	-0.43	-1.20	0.16	0.17	34	2.4	-0.88	-1.96	0.02	0.08
fixation intervall	39	0	0.15	-0.37	0.68	0.57	34	3.0	0.66	-0.14	1.46	0.09
overlap paradigm	39	0	0.55	-0.44	1.53	0.29	34	0	0.82	-0.89	2.53	0.28
step paradigm	39	0	-0.03	-0.61	0.55	0.91	34	0	-0.53	-1.46	0.41	0.32
target amplitude	38	0	-0.01	-0.05	0.03	0.64	34	0	-0.00	-0.09	0.06	0.91

*k* number of studies included in the meta-regression analysis, *PD* Parkinson’s disease, *HC* healthy controls, *proportion on med* proportion of PD patients tested in on medication state, *UPDRS III* part III of the Unified Parkinson’s Disease Rating Scale, *MoCA* Montreal Cognitive Assessment, *MMSE* Mini Mental State Examination, *mixed blocks* paradigm that included antisaccades and visually-guided saccades in the same task in randomized order

### Meta-Analysis of Levodopa Effect on Antisaccade Performance

**Fig. 5:**
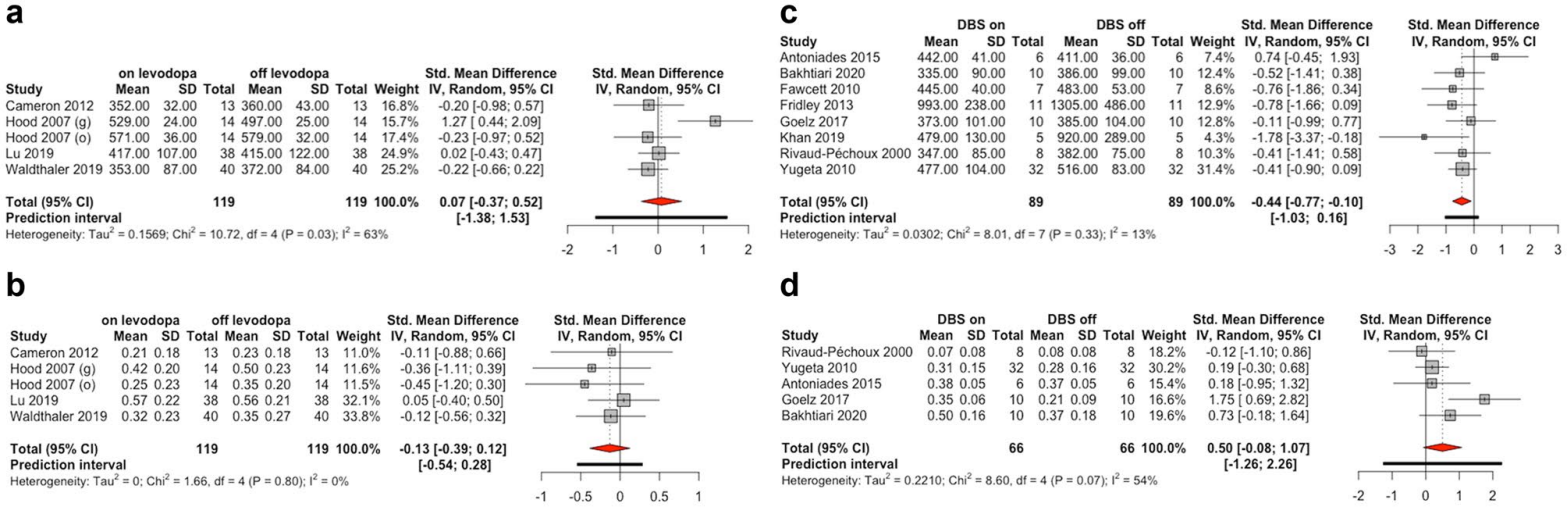
Forrest plots of the effect of levodopa (**a** and **b**) and STN-DBS (**c** and **d**) on antisaccade latency (**a** and **c**) and error rate (**b** and **d**). Negative Hedge’s g values indicate a favor for the on medication / DBS state. *CI* confidence interval, *(g)* gap paradigm, *(m)* mixed task design with prosaccades and antisaccades, *(o)* overlap paradigm, *Std.* standardized

There was no significant effect of levodopa administration on antisaccade latency ((*k*=5, g=0.07, 95%-CI =[-0.37; 0.52], *t*=0.32, *p*=0.7). or error rate ((*k*=5, g=-0.13, 95%-CI =[-0.39; 0.12], *t*=-1.01, *p*=0.3) based on the five eligible studies (Fig. 5a, b) with considerable heterogeneity (I^2^ = 62.7%, 95%-CI=[1.2%; 85.9%]; H = 1.64, 95%-CI=[1.01; 2.66]; Q(df=4)=10.72, *p*=0.03). Heterogeneity measures were low for error rates (I^2^ = 0.0%, 95%-CI=[0.0%; 49.8%]; H = 1.00, 95%-CI=[1.00; 1.41]; Q(df=4)=1.66, *p*=0.8).

### Meta-Analysis of STN DBS Effect on Antisaccade Performance

Switching STN-DBS on had a significantly reducing effect on antisaccade latency with an effect size of -0.44 (*k*=8, g=0.44, 95%-CI =[-0.7729; -0.0994] *t*=-2.54, *p*=0.01) based on 8 eligible studies (Fig. 5c) with low heterogeneity (I^2^ = 12.6% 95%-CI=[0%; 55.0%]; H = 1.07, 95%-CI=[1.00; 1.49]; Q(df=7)=8.01, p=0.3). There was a trend towards higher antisaccade error rates in DBS-on state with an effect size of 0.50, however, without reaching statistical significance based on 5 eligible studies (*k*=5, g=0.50, 95%-CI =[-1.2638; 2.2617] *t*=1.7, *p*=0.09) with considerable heterogeneity (I^2^ = 53.5% 95%-CI=[0%; 82.9%]; H = 1.47, 95%-CI=[1.00; 2.42]; Q(df=4)=8.6, *p*=0.07) (Fig. 5d).

## Discussion

This is the first meta-analysis exploring alterations of antisaccade performance in PD, which confirms significant higher antisaccade error rates and increased antisaccade latency in PD. Variables representing motor disease severity, i.e. disease duration, UPDRS III score, and Hoehn & Yahr stage mediate the effect, with a higher motor burden and disease duration being associated with prolonged antisaccade latency. Thus, antisaccade latency may provide a potential marker of disease severity in PD, as has been proposed earlier in studies with smaller sample sizes.

The antisaccade task is a well-known proxy to examine the impairment of frontal-based cognitive functions in numerous neurological diseases. Such impairment, including impaired response inhibition in the antisaccade task, is also an early symptom of PD that may even be present in the prodromal stage of the disease prior to the onset of motor symptoms. (Hogue et al., 2018) However, the dysexecutive syndrome may not be primarily caused by direct neuropathological involvement of the prefrontal cortex itself. Instead, decreased striatal dopaminergic stimulation may result in disrupted functional connectivity of fronto-striatal circuits. Early in the disease course of PD, dopamine depletion is mainly evident in the dorsolateral striatum, i.e. the caudate nucleus, which is involved in a reciprocal loop with the dorsolateral prefrontal cortex (DLPFC). (Sawamoto et al., 2008) DLPFC has been shown to play a major role in inhibiting pre-potent responses in the direction of the visual target in the antisaccade task. (Hwang et al., 2016) Importantly, other cognitive functions, that are dependent on the integrity of the dorsolateral fronto-striatal circuit, for example planning, task-switching and working memory, have been demonstrated to improve after levodopa administration. (Cools, 2006) Hence, a beneficial effect of dopaminergic medication is also conceivable for antisaccades. The separate meta-analysis of studies assessing antisaccades in off and on medication states within the same patients did not result in an overall effect of acute levodopa administration on antisaccades latency or error rate. The low number of four eligible publications was surprising, given the relatively large body of literature on antisaccade performance in PD overall.

Although phasic stimulation by levodopa administration did not have a significant effect, patients with higher chronic dopaminergic medication tended to make lower numbers of reflexive errors in the antisaccade task. If the levodopa equivalent dosage was interpreted as a proxy for disease severity in this context, the opposite association might have been expected. Instead, a potential association of higher daily dosages with lower antisaccade error rates may be interpreted as indirect evidence that levodopa may have an improving effect on antisaccade performance. However, this assumption remains speculative, since the analysis of the levodopa-equivalent daily dosage as a moderator of antisaccade error rate did not reach statistical significance.

Chronic dopaminergic stimulation may improve the dorsolateral fronto-striatal circuit in the long term and substances with long half-lives may have influenced the results of studies in off medication state. Dopamine-agonists in particular have long half-lives that may exceed the 12-hours withdrawal interval used in many studies. Furthermore, differences in the pharmacokinetic profiles of levodopa and dopamine-agonists as well as their distinct affinities for dopamine receptor subtypes may also differentially influence antisaccade performance. As such, dopamine-agonist intake may have a higher potential for increased motor response impulsivity which may result in higher antisaccade error rates. (Claassen et al., 2015) Since only a very small proportion of studies reported the exact composition of medications contributing to the levodopa equivalent daily dosage, it was not feasible to compare the effect of the different medications in this meta-analysis. More research is warranted to conclusively determine the effect of different dopaminergic drugs as well as other symptomatic medications (e.g. cholinergics, amantadine, acetylcholinesterase inhibitors) in PD. This may become particularly important in the evaluation of antisaccades latency as a marker for disease progression that would be most useful if it was not influenced by any external dopaminergic stimulation.

Until today, no study was published that reported changes of antisaccade performance in PD longitudinally. Such data would be crucial to explore the slope of antisaccade latency and error rate changes in individual patients and to evaluate the value of antisaccade latency as a marker for disease progression, for instance in upcoming clinical trials of potentially disease-modifying therapies.

As opposed to the inconclusive results regarding levodopa, STN-DBS had a significant effect on antisaccades, as switching on DBS decreased antisaccade latency which is in line with a review by Terao et al. who hypothesized that dopaminergic medication and DBS may alter saccades via distinct mechanisms. (Terao et al., 2013) That PD patients executed antisaccades faster when STN-DBS was switched on with an increased probability of errors indicates towards a shift in the speed-accuracy trade-off, i.e. an increase in motor impulsivity with STN-DBS. Generally speaking, the STN may serve as a proactive “brake” for reflexive responses of various modalities and thus, seem to play a crucial role in response inhibition. (Jahanshahi et al., 2015) In a study of local field potentials in the STN of patients with PD during DBS surgery, saccade-related beta band desynchronization during preparation of an antisaccade was stronger and longer preceding the execution of a correct antisaccade than an error. (Yugeta et al., 2013) While suppression of pathologically enhanced synchronization of beta oscillations in the sensorimotor circuits between cortex, basal ganglia and thalamus is thought to lead to the improvement of bradykinesia with STN-DBS (Little et al., 2013), the same mechanism may also result in increased motor impulsivity via the hyperdirect pathway. (Jahanshahi, 2013) Whether different locations of the DBS electrodes within STN as well as other DBS targets, such as GPi, may result in variable effects on antisaccade performance, should be investigated in future research.

Given that the course of PD and many of its symptoms are very heterogenous (Fereshtehnejad et al., 2017), the disease burden may vary substantially not only between studies, but also between subjects within the cohort. Presence and progression of cognitive impairment especially, which are highly variable between patients, have a huge impact on the overall long-term outcome. (Aarsland et al., 2004) A major limitation of this meta-analysis is that the effect of cognitive impairment on antisaccade performance could not be sufficiently evaluated based on the published literature. While several studies have provided robust evidence that antisaccade error rate is associated with executive dysfunction in healthy individuals (Hellmuth et al., 2012) and PD patients (Ouerfelli-Ethier et al., 2018), global cognitive scores did not moderate the effect of PD on antisaccade error rate or latency in this meta-analysis. MoCA and MMSE were reported infrequently in 24%, respectively 43% of the studies and many of those included varying cut-offs to exclude patients with cognitive impairment. Moreover, 61% of studies excluded patients with signs of dementia using various different diagnostic criteria. A ceiling effect may also serve as a potential explanation for the lack of a moderating effect of MoCA and MMSE on antisaccade measures in this meta-analysis. More studies with comprehensive neuropsychological assessment may provide deeper understanding of correlations between dysfunction in different cognitive domains and their consequences on antisaccades in PD.

The criteria and rigor by which matching healthy control subjects are selected might not only influence the effect size of a study but is also an indicator of its quality. In our moderator analysis, neither the group difference in age nor in the MMSE score moderated the effect of PD on the antisaccade measures. However, the validity of the finding is limited by the low proportion of 46% of studies reporting at least one cognitive score for the control group. Thus, one must assume that the majority of studies did not take measures to match subjects based on general cognitive ability or educational attainment which might have resulted in a selection bias, likely to the disadvantage of the PD group.

Although the meta-regressions did not support a moderating effect of any of the variables related to task design, the methodological heterogeneity of antisaccade studies still hampers straight-forward interpretation and comparison between studies. As shown in Table 1, study protocols have varied largely in several factors, for some of which an impact on performance has been reported. For example, mixing pro- and antisaccades within the same block adds a set shifting component to the task that may lead to an increase in antisaccade errors (Rivaud-Péchoux et al., 2007) and smaller target amplitudes may result in higher rates of express saccades, as supported by a recent meta-analysis of visually-guided saccades in PD. (Chambers & Prescott, 2010) There is an obvious need for standardization of study protocols, not only in the field of PD research. Antoniades et al. proposed a standardized antisaccade protocol in 2013 (Antoniades et al., 2013) and we encourage researchers to be guided by this proposal.

Future studies on antisaccades in PD should include sufficient clinical characteristics to ensure quality and comparability between studies, among which disease duration, levodopa equivalent dosage, a global cognitive screening score (preferably MoCA (Hoops et al., 2009)), and Hoehn & Yahr stage or, preferably UPDRS as a measure of motor disease severity may be minimum requirements. Furthermore, we want to emphasize the crucial role for careful selection of appropriate, task-naive control subjects.

In summary, this meta-analysis confirms altered antisaccade performance in PD with prolonged latency and increased error rates on a group level. While levodopa did not have a significant effect on antisaccades in a relatively small number of eligible studies, higher dosages of chronic dopaminergic medication may have a beneficial effect on antisaccade errors. In contrast, STN-DBS results in a speed accuracy trade-off, which may be indicative of increased motor impulsivity following STN-DBS in PD. The usefulness of antisaccade latency as a promising marker of motor severity in PD that may signify disease progression has to be further evaluated in longitudinal studies.

## Supplementary Information

Below is the link to the electronic supplementary material.

Supplementary file1 (PNG 268 KB)
