# Supplementary figures and images for: Antisaccades in Parkinson’s Disease: A Meta-Analysis
Source: Neuropsychol Rev. 2021 Mar 19;31(4):628–42. doi: 10.1007/s11065-021-09489-1 (PMC8592977; doi:10.1007/s11065-021-09489-1)

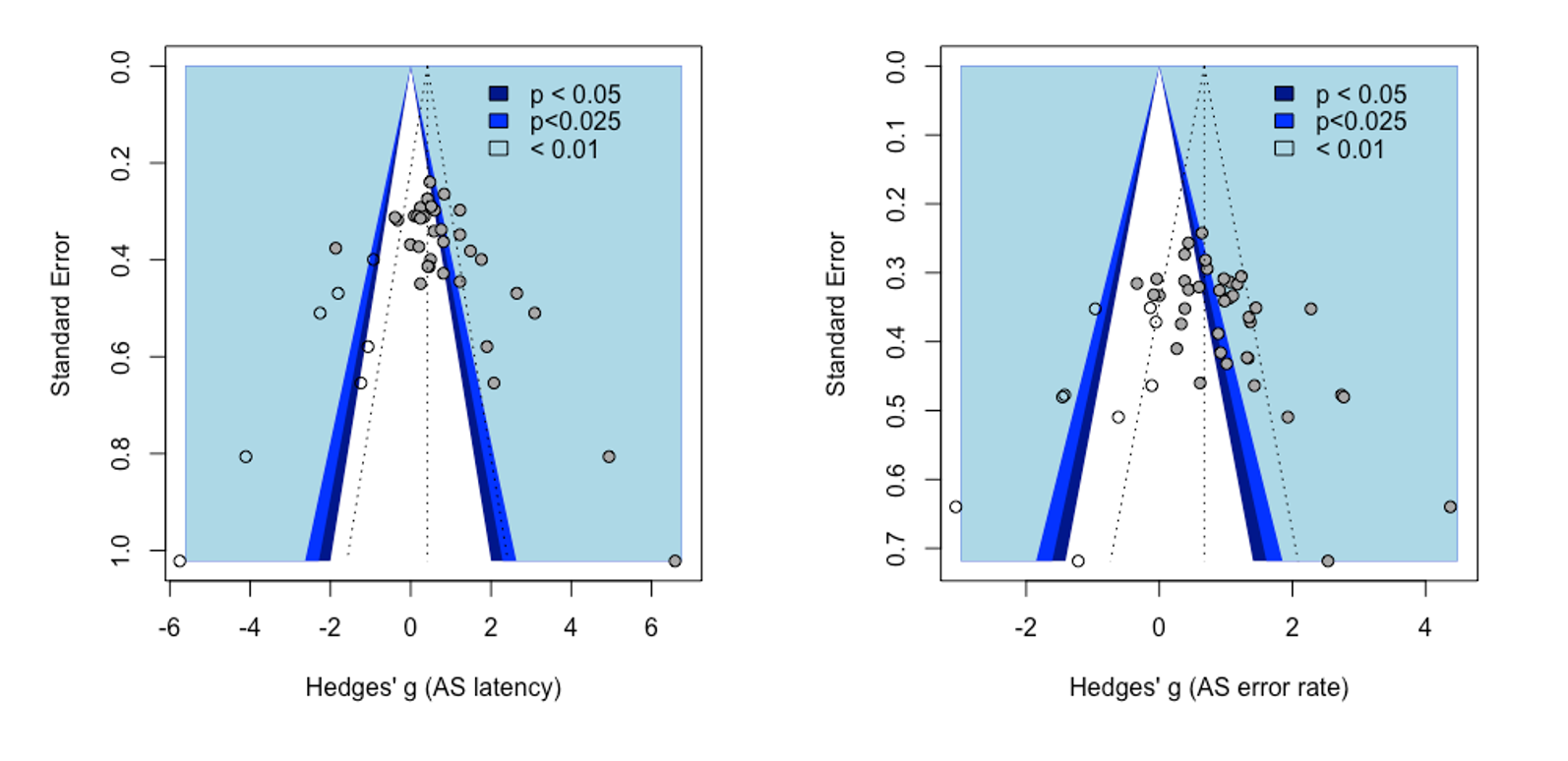

Supplement: Supplementary file 1 — Supplementary file1 (PNG 268 KB) [file 11065_2021_9489_MOESM1_ESM.png]
